# Non-Lynch Familial and Early-Onset Colorectal Cancer Explained by Accumulation of Low-Risk Genetic Variants

**DOI:** 10.3390/cancers13153857

**Published:** 2021-07-31

**Authors:** Pilar Mur, Nuria Bonifaci, Anna Díez-Villanueva, Elisabet Munté, Maria Henar Alonso, Mireia Obón-Santacana, Gemma Aiza, Matilde Navarro, Virginia Piñol, Joan Brunet, Ian Tomlinson, Gabriel Capellá, Victor Moreno, Laura Valle

**Affiliations:** 1 Hereditary Cancer Program, Catalan Institute of Oncology, 08908 Barcelona, Spain; pmur@idibell.cat (P.M.); nbonifaci@idibell.cat (N.B.); emunte@idibell.cat (E.M.); gaiza@idibell.cat (G.A.); mnavarrogarcia@iconcologia.net (M.N.); jbrunet@iconcologia.net (J.B.); gcapella@idibell.cat (G.C.); 2 Oncobell Program, Bellvitge Biomedical Research Institute (IDIBELL), 08908 Barcelona, Spain; adiez@iconcologia.net (A.D.-V.); mhalonso@iconcologia.net (M.H.A.); mobon@idibell.cat (M.O.-S.); 3 Centro de Investigación Biomédica en Red de Cáncer (CIBERONC), 28029 Madrid, Spain; 4 Unit of Biomarkers and Susceptibility, Oncology Data Analytics Program (ODAP), Catalan Institute of Oncology, IDIBELL, 08908 Barcelona, Spain; 5 Consortium for Biomedical Research in Epidemiology and Public Health (CIBERESP), 28029 Madrid, Spain; 6 Gastroenterology Unit, Hospital Universitario de Girona Dr Josep Trueta, 17007 Girona, Spain; vpinol.girona.ics@gencat.cat; 7 School of Medicine, University of Girona, 17071 Girona, Spain; 8 Catalan Institute of Oncology, IDIBGi, 17007 Girona, Spain; 9 Edinburgh Cancer Research Centre, IGMM, University of Edinburgh, Edinburgh EH4 2XR, UK; ian.tomlinson@igmm.ed.ac.uk; 10 Department of Clinical Sciences, Faculty of Medicine, University of Barcelona, 08907 Barcelona, Spain

**Keywords:** hereditary colorectal cancer, cancer predisposition, serrated polyposis, polygenic risk score

## Abstract

**Simple Summary:**

A relevant proportion of colorectal cancer patients diagnosed at young age and/or with family history of that type of cancer do not carry germline mutations in know hereditary cancer genes. Moreover, studies aimed to identify additional high-risk colorectal cancer genes were either unsuccessful or identified genes that explain extremely few cases. We aimed to evaluate the role of the accumulation of colorectal cancer low-risk variants in familial and early-onset colorectal cancer patients. We observed that the accumulation of low-risk variants may explain a relevant number of these cases, particularly in the presence of family history of colorectal cancer and of the personal history of multiple colorectal cancers. If validated in other series of patients, the identification of familial/early-onset colorectal cancer patients with accumulation of low-risk variants will translate into personalized clinical management and to the identification of additional at-risk family members.

**Abstract:**

A large proportion of familial and/or early-onset cancer patients do not carry pathogenic variants in known cancer predisposing genes. We aimed to assess the contribution of previously validated low-risk colorectal cancer (CRC) alleles to familial/early-onset CRC (fCRC) and to serrated polyposis. We estimated the association of CRC with a 92-variant-based weighted polygenic risk score (wPRS) using 417 fCRC patients, 80 serrated polyposis patients, 1077 hospital-based incident CRC patients, and 1642 controls. The mean wPRS was significantly higher in fCRC than in controls or sporadic CRC patients. fCRC patients in the highest (20th) wPRS quantile were at four-fold greater CRC risk than those in the middle quantile (10th). Compared to low-wPRS fCRC, a higher number of high-wPRS fCRC patients had developed multiple primary CRCs, had CRC family history, and were diagnosed at age ≥50. No association with wPRS was observed for serrated polyposis. In conclusion, a relevant proportion of mismatch repair (MMR)-proficient fCRC cases might be explained by the accumulation of low-risk CRC alleles. Validation in independent cohorts and development of predictive models that include polygenic risk score (PRS) data and other CRC predisposing factors will determine the implementation of PRS into genetic testing and counselling in familial and early-onset CRC.

## 1. Introduction

Genetic predisposition to colorectal cancer (CRC) may be caused by germline pathogenic variants in high penetrance genes. Germline genetic and epigenetic alterations in the DNA mismatch repair genes *MLH1*, *MSH2*, *MSH6,* and *PMS2* predispose to nonpolyposis CRC, endometrial cancer, and other tumor types. Additionally, germline heterozygous pathogenic variants in *RPS20* are a rare cause of hereditary nonpolyposis CRC. Germline pathogenic variants in *APC* and in the exonuclease domain of polymerases ε (*POLE*) and δ (*POLD1*) cause autosomal dominant adenomatous polyposis, increased risk to CRC, and to other cancers in the case of the polymerase proofreading-associated syndrome. Biallelic pathogenic variants in *MUTYH*, *NTHL1*, *MSH3,* and *MLH3* cause recessive cancer syndromes characterized by adenomatous polyposis and increased risk to CRC. In addition, *NTHL1* biallelic pathogenic variants predispose to multiple tumor types. Duplications in the 5’ regulatory region of *GREM1* cause mixed polyposis, and germline pathogenic variants in *STK11*, *BMPR1A*, *SMAD4* and *PTEN* predispose to different forms of hamartomatous polyposis [1]. Serrated polyposis (SP) is also a CRC-predisposing disease. However, except for germline heterozygous mutations in *RNF43*, which explain a very small number of cases, its genetic cause remains unexplained [2]. 

A large proportion of CRC families, mostly with nonpolyposis CRC and SP phenotypes, do not harbor pathogenic variants in known cancer-predisposing genes. Their clinical management is thus conducted based on their family history. Important efforts have been made to identify new high or moderate penetrance genes that explain the familial aggregation, early ages of onset, or polyposis phenotypes observed in those families or individuals, but the success achieved has been minimal [3,4,5].

Over the past two decades, genome-wide association studies (GWAS) for sporadic CRC have identified numerous independent association signals, which today include approximately 140 variants [6,7,8,9,10,11,12,13,14,15,16,17,18,19,20,21,22,23,24,25,26,27,28]. In 2019, Huyghe and collaborators performed low-coverage whole-genome sequencing in 1439 CRC cases and 720 controls, allowing haplotype phasing for 31.8 million genetic variants (including 1.7 million short indels and uncommon (0.1% < minor allele frequency (MAF) < 1%) variants). Moreover, they performed a meta-analysis incorporating GWAS results from >125,000 individuals, bringing the number of known independent signals for CRC to 95. The authors demonstrated that the use of a polygenic risk score (PRS) generated from the 95 association signals could impact clinical decisions for preventive screening in European populations. They estimated a familial relative risk explained by the 95 GWAS-identified variants of 11.2% (95% confidence interval (CI): 10.5–12.0), and their heritability analyses suggested that the risk to CRC is highly polygenic [24].

Recently, Archambault et al. evaluated in >12,000 early-onset CRC patients (age at diagnosis < 50) and in >95,000 CRC patients (age at diagnosis ≥ 50), the association of CRC risk with a weighted PRS, based on the 95 variants validated by Huyghe et al. They found that higher PRS was more strongly associated with early-onset CRC than with late-onset cancer, particularly in absence of family history of CRC [29]. Several years ago, in the advent of GWAS, several groups showed that increasing numbers of risk alleles were associated with familial aggregation of colorectal cancer [8,11,30,31,32].

Here, we aim to evaluate whether the validated 95 risk alleles explain the increased risk of CRC in non-syndromic nonpolyposis CRC families, where no mutations in known high-penetrance genes have been identified, and whether they are the cause of the increased CRC risk observed in SP patients. 

## 2. Materials and Methods

### 2.1. Study Participants

A total of 417 familial/early-onset mismatch repair (MMR)-proficient unrelated CRC patients (from herein on, fCRC) were included in the study (Table 1). Fifty-six (13.4%) were from families that fulfilled the Amsterdam criteria, and three hundred and sixty-one (86.6%) from families that fulfilled the revised Bethesda guidelines for hereditary nonpolyposis CRC. Hereditary CRC clinical criteria (Amsterdam criteria and Bethesda guidelines) are described in Table S1. All patients had been diagnosed with CRC and 7.7% had developed multiple primary tumors. The mean age at cancer diagnosis was 48.7 (range: 16–82). All cases showed a lack of tumor MMR deficiency and no germline pathogenic variants in the exonuclease domains of *POLE* and *POLD1* or biallelic pathogenic variants in *MUTYH* or *NTHL1*.

Eighty unrelated patients affected with hyperplastic/serrated polyposis (SP) were included in the study. Thirty-two (40%) fulfilled the World Health Organization (WHO) revised criterion I, and forty-eight (60%), criterion II for serrated polyposis [33] (Table 1). The mean age at SP diagnosis was 52 (range: 8–75). Thirty-three (41%) had developed CRC at the time of assessment, with a mean age at cancer diagnosis of fifty-three (range: 24–75). None of the patients carried germline pathogenic variants in *RNF43*.

Family history of cancer or CRC was considered when one or more first or second-degree relatives had been diagnosed with the disease. Samples and clinical data from the familial/early onset CRC and serrated polyposis patients were obtained at the Hereditary Cancer Program of the Catalan Institute of Oncology (Spain).

A total of 1077 hospital-based incident CRC patients, including cases previously analyzed in case-control studies with pathological verification and GWAS data (CRCGEN, Spain) [34], plus other consecutively recruited cases diagnosed in the same hospital, were analyzed (herein referred to as “sporadic CRC patients”). The mean age at cancer diagnosis for the sporadic cases included in the study was 67, including 74 (7%) patients diagnosed before age 50 (Table 1). A total of 1642 CRC-unaffected individuals were studied, which included controls of the CRCGEN study, plus a cohort of individuals participating in a population-based CRC screening program, most of them with a positive fecal immunochemical test (FIT) result and a normal colonoscopy or at most a low-risk adenoma (from herein on called “controls”). The mean age of controls at the time of accrual was 62.4 years (Table 1). Blood DNA extracted from the sporadic and control samples was provided by the Biobank HUB-ICO-IDIBELL (PT17/0015/0024), integrated in the Spanish Biobank Network.

All study participants had been recruited from the geographical region of Catalonia (Spain) (European/Caucasian ethnicity expected in >98%). Samples were processed following standard operating procedures with the appropriate approval of IDIBELL Ethics and Scientific Committee (PR034/14). The characteristics of cases and controls are shown in Table 1. 

### 2.2. Variant Selection and Genotyping

The analyzed variants (n = 95) and associated risks were obtained from the meta-analysis for CRC risk alleles performed by Huyghe et al. [24] (Table S2). The 95 CRC risk-associated variants reached independent genome-wide significance (*p* < 5 × 10^−8^) in large-scale GWAS as of 2019. Blood DNA samples were genotyped with the Illumina Global Screening Array-24 v2.0 designed by the Global Screening Array Consortium (GSA) (https://emea.illumina.com/science/consortia/human-consortia/global-screening-consortium.html (accessed on 1 December 2020)). The array includes 700,656 genetic variants, selected based on the Phase III of the 1000 Genomes Project (1 KGP) and reference databases for different populations, as well as 9761 markers for sample tracking, determination of offspring and sample stratification. Samples from the 417 fCRC patients, the 80 SP patients, 312 sporadic CRC patients and 854 controls were genotyped at once (24 samples/array), including in each array, whenever possible, samples from the four different groups. As internal controls, 23 unique samples belonging to the Hapmap project were included in duplicate to measure the reproducibility of the experiment (technical validation of the genotyping). Genotyping was performed at CEGEN (Centro Nacional de Genotipado, Instituto de Salud Carlos III, Spain). About 71% (n = 765) of sporadic CRC patients and 48% (n = 788) of controls had been previously genotyped with the Infinium OncoArray-500K which contains 500,000 single nucleotide variants (SNVs) with a genome-wide backbone of 250,000 tag SNVs, and includes genetic variants associated with breast, colorectal, lung, ovarian, and prostate cancers plus SNVs covering ancestry, quantitative traits, pharmacogenetics, and fine-mapping of common cancer susceptibility loci. These cases had been included in the meta-analysis by Huyghe et al. [24], but they corresponded to ~1% of the total number of cases and controls. 

### 2.3. Imputation

Thirteen of the ninety-five variants of interest were included in the Illumina Global Screening Array-24 v2.0 and thirty-seven in the Ilumina Oncoarray 500K v1.0 BeadChip. The variants that were not directly genotyped by the corresponding arrays were imputed with the Michigan Imputation Server using Minimac3 algorithm (https://imputationserver.readthedocs.io/en/latest/ (accessed on 1 December 2020)) [35]. Variants with an R^2^ lower than 0.3 (considering all genotyped samples) were excluded from the PRS analysis (rs35470271, rs145364999 and rs755229494) (Table S2).

### 2.4. Polygenic Risk Score

For each participant, a weighted PRS (wPRS) was computed using the PLINK’s score function, based on the 92 CRC risk alleles (coded as 0, 1 or 2) and effect sizes as reported by Huyghe et al. [24]. These effect sizes, when applied, were corrected for the winner’s course effect [36]. Weighted PRS values were rescaled by using as constant the mean PRS value (unweighted/weighted) of controls. To allow for missing values in some SNVs, the wPRS values were proportionally rescaled according to the number of non-missing SNVs, to the mean value observed in controls.

### 2.5. Statistical Analyses

Statistical analyses and graphical representations were conducted in R version 3.6.1 (R Core Team 2017). Two-sided t-test was applied to compare the wPRS median values between groups, and chi-squared (χ^2^) to determine differences between groups for categorical data. Odds ratios (OR) and 95% confidence intervals (95% CI) were estimated using a logistic regression model, including age and sex as covariates. Sensitivity and specificity were measured using the area under the receiver operating characteristic (ROC) curve (AUC). For this model, heritability (i.e., the proportion of variance (R^2^) explained by wPRS on the liability scale) was estimated as previously described [37].

A quantile plot was performed stratifying the population according to the wPRS. Individuals were grouped into 20 quantiles of increasing wPRS. In each quantile, the OR and 95% CI were estimated referred to the median quantile (i.e., the 10th quantile). In each regression, the covariates used in the main analyses (i.e., age and gender) were also included. 

## 3. Results

The 95 CRC risk variants [24] were analyzed in 417 familial/early-onset CRC (fCRC) patients, 80 SP patients, 1077 sporadic CRC patients and 1642 controls (Methods and Table 1). Details on the variants, genotyping platforms and imputation are shown in Methods and Table S2. Imputed Variants with an R^2^ lower than 0.3 (considering all genotyped samples) were not included in the PRS analysis, which resulted in the exclusion of rs35470271, rs145364999, and rs755229494. As shown in Table S2, 61 (74.4%) of the 82 imputed variants had an R^2^ ≥ 0.75, and 79 (96.3%), ≥0.45, indicating an overall highly reliable imputation. Figure S1 shows the workflow followed in the study. 

**Figure 1:**
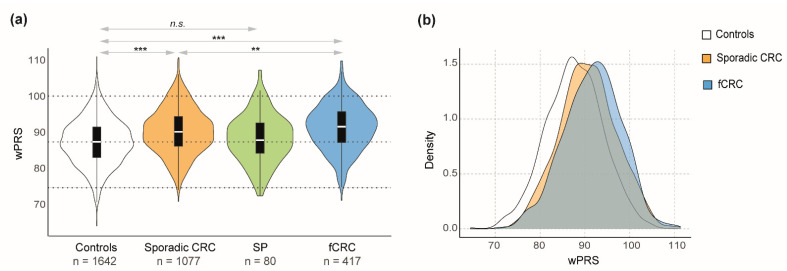
(**a**) Combined violin and box plots showing wPRS values for each group. Dotted lines indicate the median ± 2 standard deviations (SD) for controls. *** *p* < 0.001; ** *p* < 0.01; n.s. non-significant, based on *t*-test results (Table 2). (**b**) 92-variant-based wPRS plotted against the density in controls (white), sporadic CRC (orange) and fCRC (blue). Abbreviations: CRC, colorectal cancer; SP, serrated polyposis; fCRC, familial/early-onset colorectal cancer; wPRS, weighted PRS.

The distribution of wPRS among the different cohorts is shown in Figure 1. As expected, sporadic CRC patients had on average higher wPRS than controls (*p* < 0.0001; t-test) (Figure 1a; Table 2). The average wPRS was statistically higher in fCRC compared to either controls (*p* < 0.0001; *t*-test) or sporadic CRC patients (*p* = 0.004; *t*-test) (Figure 1a). In other words, wPRS was more strongly associated with fCRC when compared to controls (OR = 1.12; 95% CI: 1.09–1.14; *p* < 0.0001) and to sporadic CRC patients (OR = 1.03; 95% CI: 1.01–1.05; *p* = 0.014) (Table 2). Figure 1b shows the wPRS distribution in the different groups, highlighting the shift towards higher wPRS for fCRC patients compared to controls and sporadic CRC. Serrated polyposis patients had a mean wPRS that did not differ from that of controls, being significantly lower than the mean wPRS of sporadic CRC (*p* = 0.013; *t*-test) (Figure 1a; Table 2). SP patients affected with CRC had a wPRS similar to that of sporadic CRC patients (*p* = 0.41; *t*-test), but the difference with controls did not reach statistical significance (*p* = 0.56; *t*-test) (Table 2).

In summary, both sporadic CRC and fCRC patients had significantly higher wPRS than controls, which translated into ORs of 1.08 (95% CI: 1.06–1.09) and 1.12 (95% CI: 1.09–1.14), respectively (Table 2). Moreover, fCRC patients had, on average, higher wPRS than sporadic CRC patients (Figure 1a). No differences were observed for SP patients when compared to controls. Nevertheless, the CRC-affected SP group largely resembled the sporadic CRC group (Figure 1a; Table 2).

**Figure 2:**
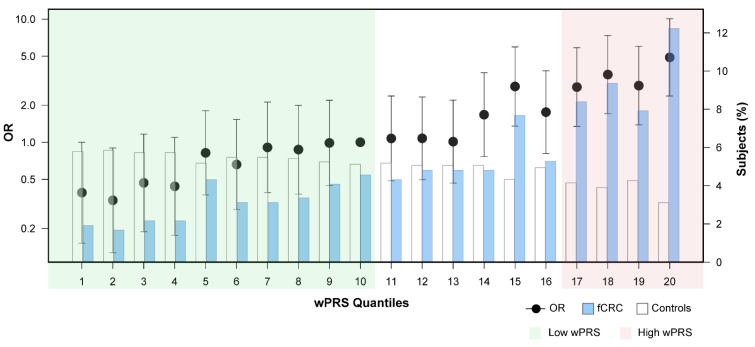
ORs (logarithmic scale) estimated for wPRS-quantile groups of fCRC patients and controls. wPRS quantiles (Q) contain near identical numbers of individuals (102–103 individuals/Q, including fCRC cases and controls). Blue and white bars represent the relative frequency of fCRC patients and controls, respectively. Q1 and Q20 contain individuals with the lowest and highest wPRS, respectively. Q10 (middle quartile) was considered the reference for OR calculations (OR = 1). Error bars indicate 95% confidence intervals. Shaded in green are low-wPRS quantiles (Q1–Q10) and, in red, high-wPRS quantiles (Q17–Q20). Abbreviations: OR, odds ratio; fCRC, familial/early-onset colorectal cancer; wPRS, weighted PRS.

Familial CRC patients in the highest wPRS quantile (20th quantile) were at a four-fold greater CRC risk than those in the middle (10th) quantile, herein considered the reference for OR calculations (OR = 4.89; 95% CI: 2.37–10.07; *p* < 0.0001) (Figure 2). We then compared the differences in demographics and clinical characteristics between fCRC patients with a high wPRS (quantiles 17–20, n = 158) and fCRC patients with wPRS below the reference (≤quantile 10; n = 127) (Table 3; Figure 2, shaded in red and green, respectively). Forty-one percent of high-wPRS fCRC patients had been diagnosed with CRC after age 50, compared to 26% of low-wPRS fCRC patients (41% vs. 26% (p_χ2_ = 0.010); OR = 1.96 (1.18–3.27), *p* = 0.00954). The frequency of cases with positive family history of CRC was higher in high-wPRS patients than in low-wPRS patients (58.4% vs. 42.7% (p_χ2_ = 0.009); OR = 1.74 (1.07–2.84), *p* = 0.0251). More high-wPRS fCRC patients had developed multiple (synchronous or metachronous) colorectal malignant tumors than low-wPRS patients (12% vs. 4.7% (p_χ2_ = 0.030); OR = 2.53 (0.93–6.85), *p* = 0.0675). No differences were observed between the two patient groups when considering gender, deceased status, or Amsterdam/Bethesda criteria fulfilment. 

The differences detected in age at diagnosis and presence of multiple primary colorectal malignancies when comparing the two PRS groups (high vs. low) were also observed without dichotomizing the patients into high and low PRS (Table S3), namely when comparing the risk, based on wPRS, according to the clinical and demographic characteristics of the patients. No differences were observed in either multiple tumor diagnosis or family history of cancer when including any type of malignancy (CRC and extracolonic tumors).

We then analyzed the interaction of wPRS with age and with CRC family history, which a priori indicated no differences in wPRS according to age groups (≤50 vs. >50; p__interaction_ = 0.125) or CRC family history (yes vs. no; p__interaction_ = 0.16). Nevertheless, these results should be taken with caution due to the limited sample size. The interaction with multiple CRCs could not be analyzed due to the small number of positive cases (<8% of fCRC). 

**Figure 3:**
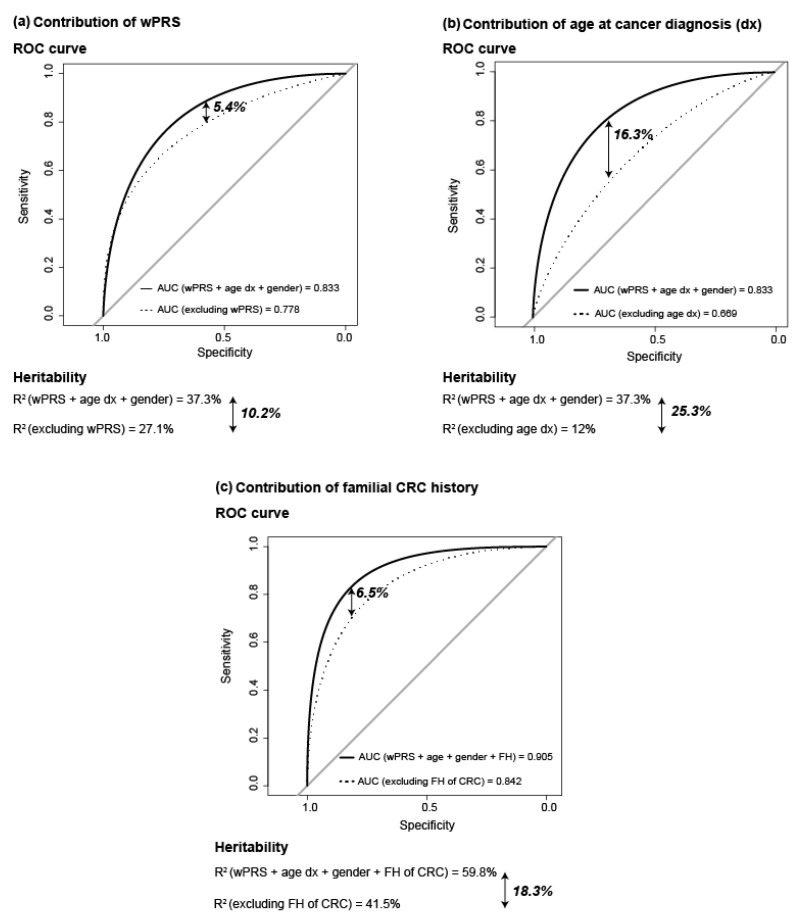
Contribution to Area Under the Curve (AUC) and heritability (R^2^) of: (**a**) 92-variant wPRS; (**b**) age at cancer diagnosis; and (**c**) familial CRC history. Data from 417 fCRC patients and 1642 controls were considered for (**a**,**b**) calculations. Due to data availability, data from 405 fCRC and 1094 controls were considered for (**c**) calculations. Abbreviations: AUC, area under the ROC curve; age dx, age at diagnosis; ROC, receiver operating characteristic; wPRS, weighted PRS.

The discriminatory accuracy of the model (sensitivity/specificity) was assessed by ROC, incorporating to the model the 92-variant wPRS, age at cancer diagnosis, and gender of fCRC cases and controls. The area under the curve (AUC) was 0.833 for fCRC patients (n = 417) compared to controls (n = 1642). AUC excluding wPRS was 0.778, and excluding age at cancer diagnosis, 0.669. This indicated that the highest contribution to the predictive model was provided by age at cancer diagnosis (contribution to AUC: 16.3%), followed by wPRS (5.4%) (Figure 3a,b). 

Knowing that fCRC corresponds per se to a highly selected population—mainly based on cancer family history and/or young age at cancer diagnosis, we also estimated the contribution of CRC family history to the model. Familial cancer history information was available for 405 fCRC patients (97% of fCRC patients) and 1094 controls (66% of the total number of controls), therefore, due to the inclusion of less individuals in the calculations, the results (slightly) differed from the AUC calculations previously shown. Taking wPRS, age at cancer diagnosis, CRC family history and gender, the AUC was 0.905, which decreased to 0.842 when excluding from the model the CRC family history, indicating that the contribution of CRC family history to the predictive model was 6.5% (Figure 3c). The contribution of gender was, in all instances, <0.5%.

Heritability, defined as the proportion of total phenotypic variation that is due to additive genetic factors and assessed in this study by R^2^ on the liability scale [37], was 37.3% for fCRC as compared to controls, when the 92-variant-based wPRS, age at cancer diagnosis and gender were included in the analysis. R^2^ decreased to 27.1% when excluding wPRS from the calculations, and to 12% when excluding age at diagnosis. This indicates that the wPRS contributed to the heritability in 10.2%, and the age at diagnosis, 25.3% (Figure 3a,b). As occurred for the ROC predictive model, R^2^ is also determined by the family history of CRC, intrinsically inherent in the selection of fCRC patients. Therefore, we determined its contribution to R^2^, which resulted in the observation that CRC family history contributed in 18.3% to the heritability (Figure 3c). 

## 4. Discussion

We assessed the potential clinical utility for familial/early-onset CRC (fCRC) of a wPRS based on 92 validated low risk alleles for CRC. We used population controls and clinic-based (“sporadic”) CRC patients as reference groups. The association of the 92-variant wPRS with fCRC (OR = 1.12; 95% CI: 1.09–1.14) was stronger than that observed in the population-based case-control analysis (OR = 1.08; 95% CI: 1.06–1.09). Our results indicate, pending validation in other fCRC cohorts, that a relevant proportion of fCRC cases without pathogenic variants in high-risk genes may be explained by the accumulation of low-risk alleles, especially in the presence of familial CRC history. 

Based on this, it seems feasible to identify the fCRC subgroup whose genetic risk is explained by a high PRS, as to warrant the application of specific surveillance measures (regular colonoscopies) to those fCRC individuals exceeding a defined wPRS value or PRS cutoff. If our findings are validated in independent cohorts, a model or algorithm that implements PRS results, family history of CRC, occurrence of multiple CRCs, and ages at cancer onset, together with data on lifestyle risk factors, would help estimate the future risk of developing cancer. Something akin to this has been developed for breast cancer, where the calculations included in BOADICEA (Breast and Ovarian Analysis of Disease Incidence and Carrier Estimation Algorithm) and implemented via the web-based computer program CanRisk (www.canrisk.org (accessed on 1 April 2021)), may be used to calculate a woman’s lifetime risk of breast and ovarian cancer [38,39]. 

Our calculations indicate that CRC-associated variants alone account for ~10% of the heritability observed in MMR-proficient fCRC, positioned third after age at cancer onset and familial CRC history. In line with our results, Huyghe et al. estimated that the variants identified to date explain about 10% of the heritable fraction of CRC risk [24]. Except for the rarely mutated *RPS20* gene (prevalence among familial/early-onset CRC patients <0.1%) [40], no (validated) genes associated with high risk to MMR-proficient nonpolyposis CRC have been yet identified [3]. The accumulation of low-risk CRC alleles might explain the highest proportion of fCRC cases not caused by MMR genes. 

The presence of CRC family history and multiple primary CRC diagnosis occurred more frequently in fCRC patients with higher wPRS than in those with lower wPRS. The association of higher PRS with CRC family history had been already detected one decade ago by several groups when ~10 risk alleles were evaluated in familial/early-onset CRC cohorts [30,31]. To our knowledge, no additional studies in CRC patients without mutations in known hereditary cancer genes assessed through hereditary cancer clinical programs have been published to date. Archambault et al., by assessing a 95-variant PRS—the same variant set as in our study, in 12,197 and 95,865 CRC patients diagnosed before and after age 50, respectively, observed that the cumulative burden of CRC-associated variants was more strongly associated with early-onset than late-onset cancer, particularly in the absence of CRC family history [29]. According to our results, their observation does not apply to fCRC patients, where high wPRS is associated with the presence of CRC family history and later age at CRC onset (≥50 years) than low-wPRS patients. Due to the limited sample size of our study, validation in larger cohorts is needed to demonstrate this association. If validated, one plausible explanation would be that the youngest cases are explained by yet-unknown genetic, epigenetic, or environmental high-risk factors, this being the cause of the apparently inverse correlation with PRS.

It is important to understand that familial CRC cases with a low wPRS may not be at lower risk, but their increased risk is possibly caused by other genetic determinants (mono-, oligo- or polygenic), non-genetic factors, or a combination of both. Therefore, they and their relatives should be managed and counselled based to their cancer family history, as recommended by current guidelines [41].

ROC analysis of models including genotype data, age and gender, alone or in combination with CRC family history, showed a high discriminative performance (AUC: 0.83–0.90). Being aware of the inherent ascertainment bias of fCRC cases—selected based on familial cancer history and/or early-age cancer onset, we determined the added value of each variable to the AUC, detecting the highest discriminative performance for age at cancer onset, followed by familial CRC history, and wPRS. The combination of those three variables, together with the occurrence of multiple CRCs, would translate into a highly improved predictive model for risk estimations in the hereditary cancer clinical context, in the absence of pathogenic variants in known high penetrance genes (as mentioned before). For sporadic CRC, the advanced predictive models recently developed using CRC risk alleles have a discriminatory accuracy, calculated by the age- and sex-adjusted AUC, of approximately 0.65 [42]. Our data shows a similar predictive ability of the wPRS in familial/early-onset CRC patients, and its combination with family history of CRC, presence of multiple tumors, and age at CRC onset, has resulted in a predictive model with greater benefit. Moreover, the implementation of newly defined risk alleles into the wPRS will probably improve its discriminatory accuracy.

Whether the PRS value defines the molecular characteristics of the tumors developed by fCRC patients, which could condition prognosis and response to therapy, remains to be investigated. Unfortunately, lack of information on cancer progression indicators, treatment, and therapeutic response in the fCRC patients included in the study, prevented us from testing this hypothesis.

Our results indicate that the 92-variant based PRS does not contribute to serrated polyposis predisposition. Due to the limited sample size (80 patients), these results should be taken with caution. Our results do not agree with those obtained by Arnau-Collell et al., where they evaluated a 62-variant PRS in 548 asymptomatic controls and 219 SP patients [43]. This study included the 80 SP patients herein assessed. Of the 62 CRC risk alleles, only 9 matched the variants reported by Huyghe et al.; 22 if those in linkage disequilibrium (LD; R^2^ > 0.8) are also considered (Table S2). They found statistically significant association of seven CRC genetic variants with SP, only three of which were included in our study or represented by a variant in LD (rs16892766, rs704017 and rs3217810). Risk alleles not included in our analysis might be involved in SP predisposition, or a larger sample size is needed to detect an association. 

Data gathered in the past years indicate that most CRC low risk alleles are located in non-coding regions and these variants may regulate the expression of target genes by altering the transcription factor-binding motif, epigenetic modification, chromatin accessibility or 3D genome conformation. Despite the unknown biological role for most CRC GWAS variants, efforts based on transcriptome-wide association analyses, expression quantitative trait loci (eQTL) analysis, or computational methods, are helping link the variants to their target genes [28,44,45,46], for many of which their role in colorectal carcinogenesis remains to be elucidated. The biological mechanisms linking CRC-associated risk variants with target genes have been validated in the laboratory for a few regions that include 8q24 MYC [47], 8q23.3 EIF3H [48], 11q23.1 COLCA1 and COLCA2 [49], 15q13.3 GREM1 [50], 16q22.1 CDH1 [51], and 18q21.1 SMAD7 [52]. Interestingly, germline high-penetrant genetic alterations in genes such as GREM1 or CDH1 are involved in mendelian forms of hereditary gastrointestinal cancer [53,54]. Other CRC risk variants might target components of the BMP/TGF-β pathway [12], key in several forms of CRC and polyposis syndromes [1].

## 5. Conclusions

Our results suggest that a relevant proportion of MMR-proficient familial/early-onset CRC cases are explained by the accumulation of low-risk CRC alleles. These findings agree with the generally unsuccessful efforts made in the past two decades to identify the genetic cause(s) of non-Lynch hereditary nonpolyposis CRC, aimed to identify high-risk causal genes. On average, wPRS is significantly higher in fCRC cases compared to controls or to sporadic CRC patients. The presence of family history of CRC, multiple primary CRCs, and later age at CRC diagnosis occur more frequently in fCRC patients with high wPRS that in fCRC patients with low wPRS. While this study illustrates the importance of clinical applicability of the PRS, our results must be interpreted with caution and should wait for validation in other familial/early-onset CRC cohorts to be translated to the clinic. 

## Figures and Tables

**Table 1:** Characteristics of the cases and controls included in the study.

	fCRC (n = 417)	SP (n = 80)	Sporadic CRC (n = 1077)	Controls (n = 1642)
**Age**				
^a^ Mean age (SD)	48.72 (12.40)	52.29 (12.60)	66.87 (10.88)	62.40 (10.25)
Range	16–82	8–75	23–91	24–92
**Gender**				
Male	235 (55.82%)	53 (66.25%)	702 (65.18%)	835 (50.85%)
Female	186 (44.18%)	27 (33.75%)	375 (34.82%)	807 (49.15%)
Ratio male/female	1.26	1.96	1.87	1.04
**Diagnostic criteria**				
Amsterdam I/II	56 (13.43%)	-	-	-
Bethesda	361 (86.6%)	-	-	-
^b^ WHO revised criterion I	-	32 (40%)	-	-
^c^ WHO revised criterion II	-	48 (60%)	-	-
**Affected with cancer**				
CRC	417 (100%)	33 (41.25%)	1077 (100%)	-
Not affected	0 (0%)	47 (58.75%)	-	1642 (100%)
**Multiple primary cancers**				
Any cancer	53 (12.70%)	11 (13.75%)	-	-
CRC	32 (7.67%)	9 (11.25%)	-	-
**Familial cancer history (1st and/or 2nd degree relatives)**				
Any cancer	334 (80.1%)	66 (82.5%)	-	136 (8.28%)
CRC	213 (51.08%)	30 (37.5%)	132 (12.26%)	84 (5.12%)
Information not available	12 (2.85%)	-	171 (15.88%)	548 (33.37%)

^a^ Mean age at cancer diagnosis for fCRC and CRC; Mean age at polyposis diagnosis for SP; Mean age at the time of blood extraction for controls. ^b^ Serrated polyposis WHO revised criterion I: at least 5 serrated polyps proximal to the rectum, all ≥5mm, with at least two ≥10mm [33]. ^c^ Serrated polyposis WHO revised criterion II: more than 20 serrated polyps of any size but distributed throughout the large bowel, with at least 5 proximal to the rectum [33]. Abbreviations: CRC, colorectal cancer; fCRC, familial/early-onset colorectal cancer; n, number; SD, standard deviation; SP, serrated polyposis; WHO, World Health Organization.

**Table 2:** Descriptive analysis of the groups according to the 92-variant-based wPRS. Each cohort is compared with the control group.

Group	N	Mean wPRS (SD)	*p* Value (*t*-Test) ^a^	OR (95% CI); *p* Value
Controls	1642	87.66 (6.37)	-	-
Sporadic CRC	1077	90.66 (6.42)	<2.20 × 10^-16^	1.08 (1.06–1.09); *p* < 2.22 × 10^-16^
fCRC	417	91.71 (6.33)	<2.20 × 10^-16^	1.12 (1.09–1.14); *p* < 2.22 × 10^-16^
SP	80	88.71 (6.65)	0.171	1.03 (0.99–1.06); *p* = 0.1536
CRC-affected SP	33	89.67 (6.70)	0.098	1.06 (0.99–1.12); *p* = 0.0562
CRC-free SP	47	88.04 (6.60)	0.699	1.01 (0.96–1.06); *p* = 0.727

^a^ Comparison of the mean wPRS value of each group vs. the control group. Abbreviations: CI, confidence interval; N, number of patients or controls; OR, odds ratio; wPRS, weighted PRS; CRC, colorectal cancer; fCRC, familial/early-onset colorectal cancer; SP, serrated polyposis; SD, standard deviation.

**Table 3:** Characteristics of high-wPRS fCRC patients (quantiles 17–20; Figure 2) compared to those with low wPRS (quantiles 1–10; Figure 2). In bold, statistically significant results.

Clinical Features	Total n (%)	High wPRS (Total n = 158)n (%)	Low wPRS (Total n = 127)n (%)	*p* Value (χ^2^ Test)	OR (95% CI); *p* Value
**Gender**					
Male Female	167 (58.60) 118 (41.40)	91 (57.59) 67 (42.40)	76 (59.84) 51 (40.15)	0.7018	1.13 (0.70–1.82); *p* = 0.6258
**Deceased status**					
Alive Deceased	260 (91.87) 23 (8.12)	144 (91.71) 13 (8.1)	116 (92.06) 10 (7.94)	0.9162	0.95 (0.39–2.30); *p* = 0.9138
**Age at first cancer diagnosis**					
<50 years old ≥50 years old	188 (65.96) 97 (34.03)	94 (59.49) 64 (40.51)	94 (74.00) 33 (26.00)	**0.0101**	**1.96 (1.18–3.27); *p* = 0.00954**
**HNPCC criteria**					
Amsterdam I/II Bethesda	41 (14.39) 244 (85.61)	24 (15.19) 134 (84.81)	17 (13.40) 110 (86.6)	0.6662	0.84 (0.42- 1.65); *p* = 0.60458
**Family history of CRC**					
Yes No	143 (51.44) 135 (48.56)	90 (58.44) 64 (41.56)	53 (42.70) 71 (57.30)	0.0092	1.74 (1.07–2.84); *p* = 0.0251
**Multiple primary CRCs**					
Yes No	25 (8.77) 260 (91.23)	19 (12.02) 139 (87.97)	6 (4.70) 121 (95.30)	0.0303	2.53 (0.93–6.85); *p* = 0.0675
**Multiple primary cancers (of any type)**					
YesNo	42 (14.74)243 (85.26)	28 (17.72)130 (82.28)	14 (11.00)113 (89.00)	0.1129	1.52 (0.73–3.12); *p* = 0.2597

Abbreviations: HNPCC, hereditary nonpolyposis colorectal cancer; wPRS, weighted PRS; CI, confidence interval; OR, odds ratio; CRC, colorectal cancer.

## Data Availability

All data relevant to the study are included in the article or as supplementary information. Additional data used and/or analyzed during the current study are available from the corresponding authors upon reasonable request.

## Supplementary Materials

The following are available online at https://www.mdpi.com/article/10.3390/cancers13153857/s1. Table S1: Amsterdam criteria and Bethesda guidelines; Table S2: 95 CRC risk-associated variants obtained from the meta-analysis for CRC risk alleles performed by Huyghe et al. and used in this study; and Table S3: Risk estimates (not adjusted by age or gender) for fCRC cases associated with the 92 CRC susceptibility alleles (wPRS). Figure S1: Strategy followed in the study.
