# Supplementary material for: Non-Lynch Familial and Early-Onset Colorectal Cancer Explained by Accumulation of Low-Risk Genetic Variants
Source: Cancers (Basel). 2021 Jul 31;13(15):3857. doi: 10.3390/cancers13153857 (PMC8345397; doi:10.3390/cancers13153857)

SUPPLEMENTARY MATERIAL

Table S1. Amsterdam criteria and Bethesda guidelines.

**Amsterdam I criteria (all criteria must be fulfilled)**<br>• There are at least three relatives affected with CRC<br>• One affected person is a first-degree relative of the other two<br>• At least two successive generations are affected<br>• At least one person was diagnosed before the age of 50 years<br>• Familial adenomatous polyposis has been excluded<br>• Tumors have been verified by pathologic examination
**Amsterdam II criteria (all criteria must be fulfilled)**<br>• There are at least three relatives with an HNPCC-associated cancer (large bowel, endometrium, small bowel, ureter, or renal pelvis, though not including stomach, ovary, brain, bladder, or skin)<br>• One affected person is a first-degree relative of the other two<br>• At least two successive generations are affected<br>• At least one person was diagnosed before the age of 50 years<br>• Familial adenomatous polyposis has been excluded<br>• Tumors have been verified by pathologic examination
**Modified Bethesda guidelines (at least one guideline must be fulfilled)**<br>• CRC < age 50 years<br>• Synchronous or metachronous colorectal or other HNPCC-associated tumors regardless of age<br>• CRC with MSI-positive morphology < age 60 years<br>• CRC with one or more first-degree relatives with CRC or other HNPCC-related tumor, with one of the cancers < age 50 years<br>• CRC with two or more first- or second-degree relatives with CRC or other HNPCC-related tumor (endometrial, stomach, ovarian, cervical, esophageal, leukemia, thyroid, bladder, ureter and renal pelvis, biliary tract, small bowel, breast, pancreas, liver, larynx, bronchus, lung, and brain (glioblastoma), sebaceous gland adenomas, and keratoacanthomas), regardless of age.

Abbreviations: CRC, colorectal cancer; HNPCC, hereditary nonpolyposis colon cancer; MSI, microsatellite instability.

Table S2. 95 CRC risk-associated variants obtained from the meta-analysis for CRC risk alleles performed by Huyghe et al. and used in this study.

[Included as separate file]

Table S3. Risk estimates (not adjusted by age or gender) for fCRC cases associated with the 92 CRC susceptibility alleles (wPRS). In bold, statistically significant results.

Clinical features	fCRC patients (%)	Mean wPRS (SD)	OR (95% CI)	*P* value
Gender				
Male	232 (55.63)	91.59 (6.62)		
Female	185 (44.36)	91.87 (5.96)	1.008 (0.977-1.039)	0.6278
Deceased status				
Alive	380 (91.12)	91.66 (6.39)		
Deceased	33 (7.91)	92.04 (5.77)	1.008 (0.953-1.066)	0.7879
Age at diagnosis				
<50years	271 (64.98)	91.18 (6.68)		
≥50years	146 (35.01)	92.70 (5.51)	**1.038 (1.005-1.072)**	**0.0252**
HNPCC criteria				
Amsterdam I/II	56 (13.43)	91.90 (5.83)		
Bethesda	361 (86.57)	91.68 (6.41)	0.994 (0.951-1.039)	0.7961
FH CRC				
No	192 (46.04)	91.06 (6.64)		
Yes	213 (51.07)	92.28 (6.09)	1.030 (0.999-1.063)	0.0575
FH cancer				
No	71 (17.02)	91.59 (6.69)		
Yes	334 (80.09)	91.72 (6.32)	1.003 (0.964-1.044)	0.8760
Multiple CRC				
No	385 (81.74)	91.47 (6.25)		
Yes	32 (7.67)	94.65 (6.64)	**1.088 (1.024-1.156)**	**0.0067**
Multiple any cancer				
No	364 (87.29)	91.42 (6.25)		
Yes	53 (12.71)	93.69 (6.55)	**1.06 (1.011-1.112)**	**0.0160**

Abbreviations: CI, confidence interval; CRC, colorectal cancer; fCRC, familial/early-onset CRC; FH, family history; HNPCC, hereditary nonpolyposis colorectal cancer; OR, odds ratio; SD, standard deviation; wPRS, weighted polygenic risk score.

Figure S1. Strategy followed in the study.

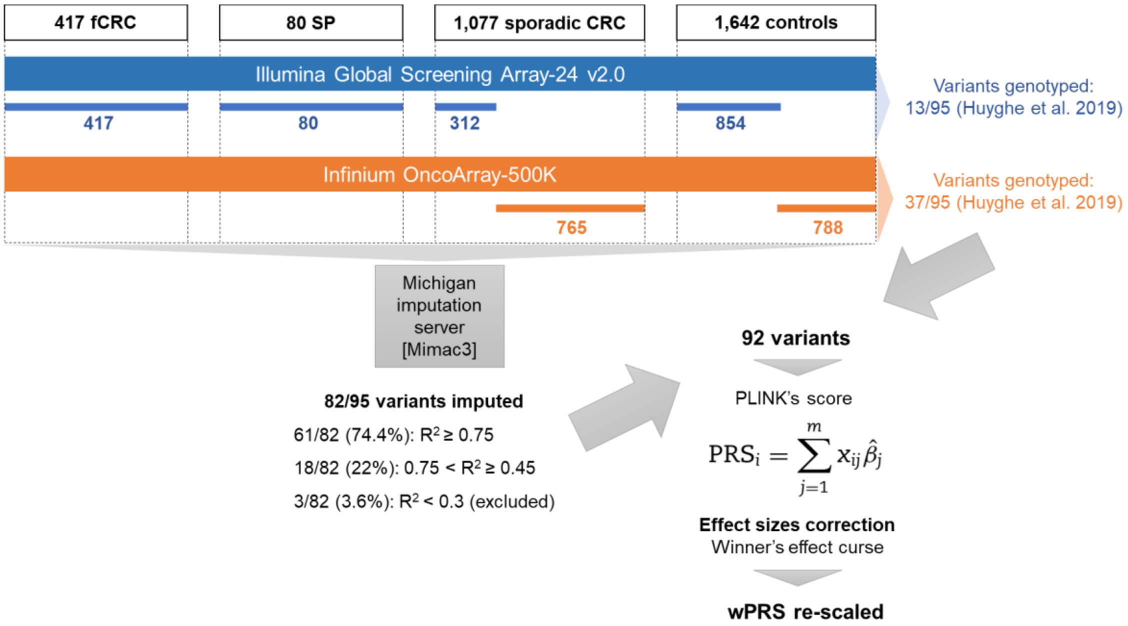

Supplement: Supplementary file 1 [file cancers-13-03857-s001.zip › Suppl. Material.pdf]
